# Medical Students’ Attitudes Toward a Career in Psychiatry: A Realist Evaluation

**DOI:** 10.1007/s40596-025-02125-7

**Published:** 2025-03-21

**Authors:** Lucy Hollands, Elizabeth McCulloch, Jessica Scott, Jason Hancock, Karen Mattick

**Affiliations:** 1 University of Exeter, Exeter, UK; 2 Devon Partnership NHS Trust, Exeter, UK; 3 Faculty of Health and Life Sciences, University of Exeter, Exeter, UK

**Keywords:** Medical students, Attitudes, Career, Psychiatry

## Abstract

**Objective:**

There is a global recruitment crisis in psychiatry, with insufficient medical students choosing this specialty as their medical career. Previous research describes multiple interacting factors that may influence medical students’ attitudes toward a psychiatry career but not which are most important for whom, in what respects, and in what contexts, nor how they interact to shape attitudes. Therefore, this study aimed to develop a theory to explain the complex factors shaping medical students’ attitudes toward a psychiatry career.

**Methods:**

This realist evaluation involved three phases: phase 1 was the development of an initial program theory from published literature; in phase 2, a final program theory was developed from realist interviews with 31 UK medical students; in phase 3, evidence-based recommendations were developed.

**Results:**

This study identified seven important contexts (positive personal experiences of mental health services; exposure to multiple psychiatry subspecialties as undergraduates; positive experiences on psychiatry placement; perceived good work-life balance for psychiatrists; perceived danger from psychiatric patients; perceived poor patient prognoses; perceived negative emotions evoked through work) and six important mechanisms (being inspired to make a difference; being interested in psychiatry; seeing psychiatry as aligned with desired lifestyle; being concerned about safety; feeling unable to make a difference; being concerned about emotional burden). These contexts and mechanisms interacted to shape positive or negative attitudes toward a psychiatry career.

**Conclusions:**

Based on the findings, the authors recommend more pre-clinical psychiatry placements, exposure to passionate psychiatry teachers especially on psychiatry placements, and a focus on retention of interested students.

Health care globally is facing a human resource crisis, including challenges with both recruitment and retention. The worldwide shortage of skilled health care professionals is predicted to be 12.9 million by 2035 [1]. Psychiatry continues to face workforce shortages in many countries [2], for example, with a 10.7% vacancy rate in the UK’s National Health Service (NHS) in 2021 [3]. This is also reflected in many other developed and developing countries [4, 5].

In the UK, from 2016 to 2019, there was a 21% increase in people seeking mental health service support [6]. This has been further compounded by COVID-19, with over 1.4 million people on mental health waiting lists in 2021 [7]. Taken together, increasing patient load combined with a staffing shortage may lead to existing staff members feeling overworked and burned out, which may lead them to exit the field of psychiatry.

Professional bodies internationally have been seeking solutions to address this recruitment and retention crisis. In the UK, the Royal College of Psychiatrists (RCPsych) created and implemented a “Choose Psychiatry” campaign aimed at recruiting medical students (through many strategies, including promotional videos and medical school engagement) [8]. The European Psychiatric Association developed guidance on improving the image of psychiatry, and the European Federation of Psychiatric Trainees recognized the importance of promoting positive image in recruitment [8]. Such strategies to address recruitment and retention have been encouraged by the World Psychiatric Association [2].

In recent years, there is evidence of an improvement in recruitment into postgraduate psychiatry training. In the USA, rates of matching into psychiatry resident programs have increased for the last 13 years [9], while in the UK there has been an increase in the proportion of training posts filled since the start of the “Choose Psychiatry” campaign [3]. Despite these attempts to improve recruitment in psychiatry, vacancies still persist [2–5]. To fill these training positions, medical students must choose to undertake a career in psychiatry. Currently, only 3.6% of UK medical students are pursuing a career in psychiatry, whereas about 6% are needed to meet future patient demand [10]. For the purposes of this study, we define a medical career as the choices made that alters career trajectory with regard to training progression and specialty choice. The theory of planned behavior suggests that attitudes influence intentions, which, in turn, inform behaviors [11]. Therefore, medical students’ attitudes toward psychiatry are critical in determining the choice of psychiatry as a career. We chose to focus on medical students’ attitudes, reasoning that intervention early in medical careers (e.g., as undergraduates) might offer the most potential to change behaviors at the point of specialty choice.

Medical students’ attitudes toward a career in psychiatry have been studied worldwide. Unfortunately, views are often negative: studies internationally have identified that students perceive psychiatric patients to be complex, with a high morbidity and poor prognosis, potentially dangerous, and associated with emotional burden [12–16]. Additionally, students often believe psychiatrists earn less money, work longer hours, and are less respected than non-psychiatric faculty [17–20]. Students also cite psychiatry as a career with low prestige and little scientific basis [12–14].

Despite this, it is reported that positive attitudes may develop if students have longer, more positive, or more varied experiences on placement in psychiatry [21–24]. Personal factors, including female gender, a family history of mental illness, family members working in psychiatry, and a background in humanities, are also associated with more positive attitudes toward a career in psychiatry [25–28]. Finally, psychiatry is viewed positively as a career with the potential to work less than full time [29].

In summary, previous research has described multiple interacting factors that may influence medical students’ attitudes toward a psychiatry career, but we do not know how they interact to shape attitudes in different contexts. Therefore, this study aimed to develop a theory to explain the complex factors shaping medical students’ attitudes toward a psychiatry career.

## Methods

This study aimed to explain how medical students form positive or negative attitudes toward a career in psychiatry. We used an approach called realist evaluation to explore the contexts in which certain mechanisms are triggered to produce outcomes (see Table 1 for definitions [30]).

**Table 1:** Definitions of key terms

Key term	Definition
Realist evaluation	Type of theory-driven evaluation method used in evaluating social programs, where there are many interwoven variables operating at different levels
Program theory	Explanation of how programs produce outcomes and under what circumstances
Context	Specific aspect of the setting/environment of study, which when present triggers the activation of a mechanism(s)
Mechanism	Latent (often invisible) property/entity, sensitive to variations in context, which when activated causes an outcome to occur
Outcome	Desirable (or undesirable) event/occurrence, which is of interest by its presence or absence (or strength/degree)
Context-mechanism-outcome configuration (CMOc)	Analytical unit/statement/diagram which links and orders a context, mechanism, and outcome, to provide a causal explanation, often abbreviated to CMOc

The aim was achieved through three objectives relating to three research phases. The first objective (phase 1) was to produce an initial program theory (i.e., an initial explanation of how attitudes are formed) through a rapid review of literature. The second objective (phase 2) was to form a final program theory (i.e., a more developed explanation of how attitudes are formed) through realist interviews with medical students. The third objective (phase 3) was to create realistic and practical recommendations to improve medical students’ attitudes toward a career in psychiatry.

### Study Design

Career decision-making is complex, relying on internal and external influences that are unique to each individual [31]. Realist evaluation recognizes the challenges associated with finding solutions for complex problems by aiming to uncover “what works, for whom, in what circumstances and how?” [30]. Some of the contexts that influence medical students’ attitudes toward a career in psychiatry have already been described in the literature, so this realist evaluation captured the already known contexts, mechanisms, and outcomes, as well as discovering new ones [30].

A realist evaluation starts with an initial program theory (i.e., an initial explanation of how attitudes toward a career in psychiatry are formed) by linking contexts to mechanisms and outcomes (see Table 1) [30]. This initial program theory is then modified through a method different to the one used to create the initial program theory (e.g., a literature review may create the initial program theory and interviews or a questionnaire may develop it) to create a final program theory [30]. The methodological approach follows the Realist and Meta-narrative Evidence Synthesis: Evolving Standards 2 (RAMESES II) reporting guidelines [32] and Manzano’s approach to realist interviewing [33].

### Phase 1: Generating an Initial Program Theory

The initial program theory was created through a rapid literature review. The literature review was not intended to be exhaustive since, as is typical in realist research, it simply created a starting point for the realist interviews. MEDLINE, ERIC (Educational Resources Information Center), EMBASE (Excerpta Medica Database), and the Cochrane Database of Systematic Reviews databases were searched using specific search terms to identify literature exploring medical students’ attitudes toward a career in psychiatry. Additional literature was identified through forward and backward citation searching. Abstracts of all identified articles were screened against the inclusion criteria. Full texts were screened against the same criteria, and included papers were read in full, with relevant contexts and mechanisms extracted. Search strategy details can be provided on request.

The initial program theory included contexts, mechanisms, and outcomes identified from the included literature. As is typical in realist research, discussions among the research team, based on their real-world experience (see the “Reflexivity” section), identified additional potential contexts and mechanisms to add to the initial program theory.

### Phase 2: Realist Interviews

#### Study Context

The study setting was a 5-year undergraduate-entry medical school with approximately 1000 students in southwest England. The first 2 years of this program are spent in the academic environment with brief (half-day) clinical placements, which may be in a psychiatric setting. The final 3 years are spent in clinical environments with formalized psychiatry placements taking place in year 3 (3 weeks) and year 4 (2 weeks). Final-year students have longer placements (6 weeks), which may include psychiatric placements, but they are not compulsory. While variation exists nationally, this is broadly consistent with all UK medical schools. Following graduation, doctors spend the first 2 years of training in a foundation training program, typically six placements of 4 months. One of these may be in a psychiatry setting. Formal postgraduate psychiatry training begins after completing the foundation program. Therefore, decisions regarding an application into psychiatry training programs typically formally take place during the second foundation year. In the UK, psychiatry training consists of 3 years of core psychiatry, experiencing different subspecialities before applying for a higher training program in a specific subspeciality.

#### Sampling and Recruitment

We used purposive sampling to maximize diverse perspectives of medical students across all 5 years of study. This sampling strategy recognized that medical students’ attitudes toward specialties can develop early in medical training and career intentions may become clear at different times during medical school. Participants did not have to have a formed decision whether they would pursue a career in psychiatry, to ensure both positive and negative viewpoints were considered. Students were invited to participate and provided with participant information through the medical school societies’ social media pages. All participants completed an informed consent form before participation.

#### Data Collection

Realist interviews were conducted by LH using Manzano’s methodological framework for realist interviewing [33]. Questions were refined through a pilot interview with a student from a different medical school. Interviews were semi-structured and took place in person or online, according to participant preference.

Interviews built upon the initial program theory by identifying the circumstances (i.e., contexts) that are likely to lead to positive or negative attitudes toward a career in psychiatry (i.e., outcomes) and the causes underlying this (i.e., mechanisms). Each interview was unique, using the developing program theory as the starting point [33]. The roles of interviewer and participant varied at different points in the interview cycle, with the interviewer sometimes taking the lead to explain parts of the program theory and describe the viewpoints of previous participants, in what realist researchers call the teacher-learner cycle [33].

Interviews changed in emphasis as the study progressed [33]. In the early interviews, open-ended, exploratory questions were asked. Participants discussed their experiences and attitudes toward a career in psychiatry and their opinions on the initial program theory, highlighting the conditions it applies to their real-world settings. In subsequent interviews, the focus remained on participant experiences, but questions were aimed at determining which connections in the evolving program theory were most important to them. In the final interviews, the connections between contexts, mechanisms, and outcomes were verified, transforming the developing program theory into a final program theory. Additionally, participants in the final interviews were asked to share their recommendations for improving students’ attitudes toward a career in psychiatry. Each interview phase comprised around 10 interviews, although, as is typical in realist research, the distinction between phases was not absolute. A topic guide illustrating the three phases of interviewing is available on request.

#### Data Analysis

The interviews were recorded, transcribed, and analyzed using NVivo version 12.4. LH led the data analysis, using both inductive (i.e., data-led) and deductive (i.e., led by the initial program theory) approaches. The program theory was then iteratively developed concurrently to data collection, resolving any disagreements through discussion by the whole author team. We sought to identify contexts, mechanisms, and outcomes within the data, and their relationships. We analyzed repeated patterns in the data to understand the specific circumstances in which they occurred and used the frequency and conviction of these to iteratively develop the program theory. These iterative cycles of refinement eventually led to a final program theory that summarized the key elements of the most important context-mechanism-outcome configurations (CMOcs).

### Phase 3: Generating Recommendations

As is common in realist research, interview participants were actively involved in suggesting ideas to improve the current situation, so the recommendations arising from this study are grounded in data. Putative recommendations were coded as part of the analysis process, discussed at research team meetings, and shared and refined in subsequent interviews for other participants to comment on their importance, feasibility, and value in real-world contexts. The final recommendations are those that gained significant support from multiple participants and are central to the final program theory.

#### Reflexivity

The lead author (LH) is a fifth-year medical student who is currently undecided about a career in psychiatry. The coauthors are a newly qualified doctor (EM), a psychiatry specialist trainee (JS), a consultant psychiatrist and senior medical educator (JH), and a professor of medical education (KM). All coauthors have experience of realist research. As is common in realist research, the diverse team perspectives (e.g., profession and career stage) were seen to enrich the analysis and decision-making, ensuring multiple perspectives were considered and maximizing the research rigor and impact potential. LH is a peer to some participants, which may have influenced the interview discussions. With JS and JH working in psychiatry, our team had unique insights into how recommendations could function in the real world. Since LH, EM, and KM do not work in psychiatry, recommendations that might not seem feasible within psychiatry currently could also be explored.

#### Research Ethics

The University of Exeter Medical School Research Ethics Committee provided ethical approval (ref 418/22/12/04). We sought to minimize risk to participants and researchers, for example, through written participant consent; anonymized data ensuring participant confidentiality; protecting and encrypting recordings, transcriptions, and data storage in line with General Data Protection Regulations; and providing detailed study reporting to ensure transparency.

## Results

### Phase 1: Developing the Initial Program Theory

**Fig. 1:**
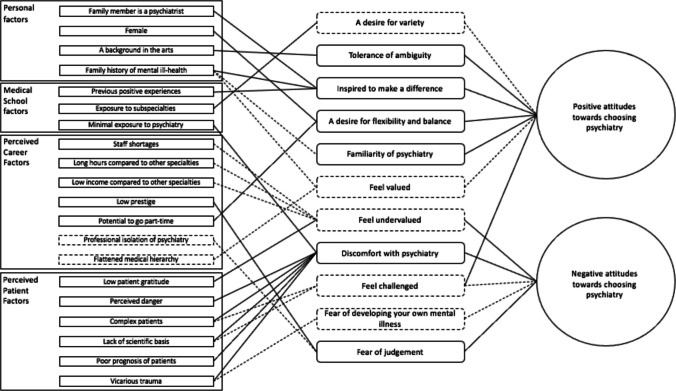
Initial program theory: rectangles represent contexts; rounded rectangles represent mechanisms; ovals represent outcomes; dashed lines represent contexts and mechanisms that emerged from authors’ discussion; arrowheads are omitted to avoid suggesting causation due to speculative nature of connections in initial program theory

An initial program theory was created as the starting point for the research, based on the contexts, mechanisms, and outcomes identified through the rapid literature review and author discussions (Fig. 1).

### Phase 2: Realist Interviews

#### Development of the Final Program Theory and CMOcs

Thirty-one medical students were interviewed (Table 2). The participant demographics are fairly representative of medical students nationally [34]. The early interviews identified 22 further contexts and eight further mechanisms, to supplement those in the initial program theory. Subsequent interview phases refined connections between context, mechanisms, and outcomes theory. Some elements were combined (e.g., “feeling valued” and “feeling needed” became a single mechanism, as participants explained these with common meaning), and some were removed (e.g., “underfunding of psychiatry” was viewed by participants as more widespread than this one specialty and, therefore, not a major context in shaping attitudes to psychiatry specifically). These changes were discussed and agreed by the full research team. The iterative process of developing the initial program theory into the final program theory is available on request.

**Table 2:** Realist interview participant demographics (*N* = 31)

Demographic	Participant *n* (%)	Intake of medical students at all UK medical schools 2021–2022 (*N* = 10,500) [47] (*n*, %)
Gender		
Male	6 (19)	3785 (36)
Female	25 (81)	6710 (64)
Other	0 (0)	5 (0)
Year of medical school		
1	3 (10)	
2	6 (19)	
3	6 (19)	
4	7 (23)	
5	9 (29)	
International student		
Yes	5 (16)	965 (9)
No	26 (84)	9535 (91)
Previous higher education experience		
Yes	1 (3)	
No	30 (93)	

**Fig. 2:**
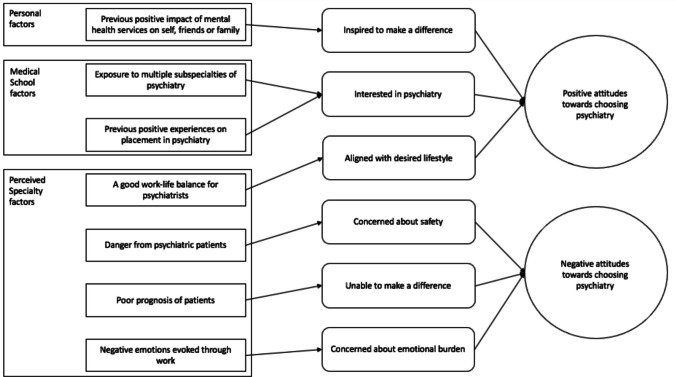
Final program theory: rectangles represent contexts; rounded rectangles represent mechanisms; ovals represent outcomes

The final program theory (Fig. 2) contains seven CMOcs (Table 3). It aims to explain the complexity of contexts, mechanisms, and their linkages that shape medical students’ attitudes toward a career in psychiatry. These CMOcs are those with the strongest supporting evidence from the interviews, and illustrative quotes are provided (Table 3). Additional illustrative quotes for each context, mechanism, and outcome (separately) are available on request.

**Table 3:** Seven context-mechanism-outcome configurations (CMOCs) identified through analysis of interview data, with illustrative quotes

CMOc	Description	Illustrative quote
1	If medical students have exposure to positive experiences of mental health services, either personally, or through friends or family (C), they may feel inspired to make a difference (M), forming positive attitudes toward a career in psychiatry (O)	Participant 19: I’m quite interested in [psychiatry], probably more so than other specialities which aren’t as interactive with the patients (O), I think from my experiences as a patient… Personally, when I’ve been treated by some psychiatrists… [they] have been really good and they’ve made me feel listened to, they acknowledged me, and they wanted to help and do everything that they could to. Also, my sister has been through quite a bit, been admitted to an inpatient place and and just I know how difficult it was for her, the difficulty she’s faced…. (C) I definitely feel like a big thing was the positive impacts and the positive experience from some particular professionals listed. It’s inspired me (M)
2	If medical students are exposed to multiple subspecialties of psychiatry (C), it can ignite an interest in psychiatry (M), developing more positive attitudes toward a career in psychiatry (O)	Participant 8: I got to realise [on placements in subspecialties] how broad psychiatry is and all of the different aspects you can cover, right the way down from child and adolescent psychiatry all the way up to general adult psychiatry and then psychiatry for older people (C). It’s really across the whole life course, so that was really interesting to me. The options seemed pretty endless and not just tied to the hospital or clinical setting, which is really interesting… (M) So, I’m really quite keen on psychiatry (O)
3	When students have positive experiences on placement in psychiatry (C), it can give students a feeling that psychiatry is interesting (M), developing more positive attitudes toward a career in psychiatry (O)	Participant 14: When I was on psychiatry, I found it interesting (M)… I found that the people working within the field were interested in and slightly more invested in our experience than in other placements…. I think it’s how you’re treated on placement or whether it was a positive experience because when you make decisions about anything, you’re basing it mostly on previous experience… If it was a more positive experience, then you’re more likely to come back to it or to consider it as a career…. (C) I would happily go into psychiatry (O)
4	Students that hold the perception that psychiatrists have a good work life balance (C) feel that psychiatry aligns with their own lifestyle desires (M), forming more positive attitudes toward a career in psychiatry (O)	Participant 3: Your lifestyle is great. It works in terms of having a family and holidays (C) and so I guess being able to see it from all aspects, not only does it fit what I want in terms of having a profession, it also fits the side of things of, actually this will be hopefully great for me in the long run financially and in terms of my lifestyle as well… I would like to think that I would have a timetable where yes, I am going to miss things, but I’m also going to have time to go to things and come home at a certain time and not have to do night shifts once I get to a certain age and also, I guess be able to enjoy having my family as well (M)…psychiatry is the best of both worlds (O)
5	A perception of danger from psychiatric patients (C) can make students concerned about their safety were they to undertake a career in psychiatry (M), forming negative attitudes toward a career in psychiatry (O)	Participant 1: I’ve done a variety of psychiatry placements and I’ve had experiences where, even as a male, I’ve felt unsafe. There are times where I’ve walked onto a psychiatry ward and the patients have been just been walking around. I’ve got no idea who the patients are. Some of their backgrounds can be quite violent and… The staff are in their offices or behind these glass screens in locked rooms and I’m on my own talking to a random psychiatric patient who may or may not be violent… some of these psychiatric patients can be quite violent and spontaneous (C) … I don’t really want to be feeling that unsafe when working… (M) so that’s sort of why I don’t want to go into the specialty (O)
6	Students with the perception that psychiatric patients have a poor prognosis (C) are concerned that they will be unable to make a difference (M), thus forming more negative attitudes toward a career in psychiatry (O)	Participant 15: I think what I find hard about psychiatry is that a lot of the time, there’s no instant solution. And for a lot of the patients, the prognosis is quite poor (C)… I guess the reason a lot of people go into medicine, including me is you want to make people better, it sounds so cliché… I just find it quite satisfying when you get that outcome…. Whereas it’s so hard when you don’t see that in psych… you feel like you’re not helping people. That’s why I chose medicine in the first place (M)… it’s probably not for me (O)
7	When students hold the perception that working psychiatry evokes negative emotions (C), they are concerned about the emotional burden they would have as a psychiatrist (M), resulting in negative attitudes toward a career in psychiatry (O)	Participant 6: I’ve always thought it must be quite personally draining being a psychiatrist (M). You’re getting an insight into a person’s mind who isn’t very well and are exposed to quite a lot upsetting details (C) and I can imagine it can be quite hard to not bring your work home and I can imagine it can take a bit of a toll… I don’t know if I do want to go into that (O)

*C*, context; *M*, mechanism; *O*, outcome

### Phase 3: Generating Recommendations

Through the research process, we identified four recommendations for stakeholders (e.g., medical faculty, professional bodies, psychiatry recruitment campaigns).

#### Ensure Medical Students Have Dedicated Mentors While on Psychiatry Placement

Medical students highlighted that dedicated mentors to guide or shadow on psychiatry placement might help students to feel safer (CMOc 5) and more supported and increase student confidence in talking to patients. This may also create more positive experiences on psychiatry placement (CMOc 3), resulting in more positive attitudes toward a career in psychiatry.

Shadowing someone gives you an actual insight into what the job involves. All of your perceived fears, which I had, can be completely gone. The doctors were so calm and collected and it really does impact you. I was a lot more open then to talk to the patients… Then you’re going to have more positive experiences because you’re going to know how to deal with it. (Participant 27)

…having someone that’s a mentor to you or maybe more dedicated to your learning, but particularly in psychiatry because psychiatry patients are quite different… If you can see a doctor or someone else talking to those patients, knowing that they’ve got to talk to those patients in order to help them, it can be really helpful to see those interactions and learn from them. (Participant 30)

#### Provide More Psychiatry Placements in Pre-clinical Years of Medical School

Participants highlighted the importance of having placements in psychiatry early in medical school. Early exposure allows medical students to consider pursuing psychiatry earlier. Additionally, this extra time allows adjustment to interactions with mental health patients, potentially reducing perceptions of danger (CMOc 5) and, therefore, negative attitudes toward a career in psychiatry. Additionally, early placement facilitates medical schools to provide exposure to different subspecialties of psychiatry (CMOc 2), which participants told us improves attitudes among medical students.

I think an important thing is early exposure to psychiatric placements during medical school. In my second year, I was lucky enough to go to the special disability unit... I was a little scared just because it’s a bit of an unknown, but the staff were lovely. I got to speak to the patients, and it was a really eye opening experience… More exposure to broad-based psychiatry in the earlier years could spark an interest, and then you can explore that with that extra time… It is really important to have really early exposure of psychiatry. (Participant 27)

#### Exposure to Passionate and Inspiring Teachers in Medical School

Participants consistently highlighted that passionate and inspiring teachers could improve attitudes toward psychiatry. Passionate and inspiring teachers may create more positive experiences on placement in psychiatry (CMOc 3). These teachers are more likely to highlight the positive aspects of a career in psychiatry, for example, a good work-life balance (CMOc 4) and discuss negative perceptions, for example, the perception of danger (CMOc 5). Additionally, passionate teachers may encourage students to understand how to cope with the negative emotions evoked through work (CMOc7), thus improving medical students’ attitudes toward a career in psychiatry.

You want to do something that you’ll enjoy and if you can see other people are enjoying doing it and are passionate about it themselves, then I feel like it makes it more likely that you think, maybe there’s something that I could be passionate about as well and could really enjoy spending my time doing. (Participant 30)

We have a really good lecturer who’s done so well in surgery. Whenever he puts on a talk, people flock to go and watch him. It does work, and of course, it inspires you because you look up to the people who have done well, and you want to aspire to be like them. (Participant 26)

#### Focus on Retention of Interested Students

Participants suggested that increasing spaces and promoting pre-existing specialty recruitment schemes may help retain students already interested in psychiatry through exposure to subspecialties (CMOc 3) and positive placement experiences (CMOc 4). Additionally, augmenting these schemes may address the negative perceptions of psychiatry, for example, the perception of danger (CMOc 5) from psychiatric patients or the perception of psychiatric patients having poor prognoses (CMOc 6).

I’ve seen a lot of the Choose Psychiatry adverts, I’ve been at conferences, and I’ve seen the effort that there is being put into recruiting medical students to do psychiatry, and I think it’s working… Opportunities of the Psych star scheme [a UK-based recruitment scheme] and psychiatry foundation fellowship and things like that are good ways of retaining that interest. (Participant 14)

I think surgical societies and career events get thrown down your throat on student Facebook pages, and even from the uni, we get e-mailed stuff all the time, like surgical research… I don’t think the uni is good at spreading things from other specialities, particularly psych. I think more awareness of psych socs [psychiatry student societies] and a bigger presence of things like that would definitely be good… Psych campaigns need to be more active on social media… a specific Facebook page for students interested in psychiatry, so it doesn’t get lost. (Participant 31)

## Discussion

This study aimed to generate a theory explaining how medical students form positive or negative attitudes toward a career in psychiatry. The final program theory demonstrates how the most important identified contexts interact to form attitudes toward a career in psychiatry. The theory included seven contexts, six mechanisms, and two outcomes, combined into seven CMOcs, highlighting the complexity that shapes medical students’ attitudes toward a career in psychiatry. Although no single CMOc is considered causal in itself, in combination, they shape an overall positive or negative attitude toward a career in psychiatry.

A growing body of literature recognizes that context influences medical students’ attitudes toward a career in psychiatry. Although previous research has explored medical students’ attitudes toward a career in psychiatry, this is the first realist evaluation exploring this subject. It is clear that the influences on medical students’ attitudes toward a career in psychiatry are multiple, dynamic, and interacting, which plays to the strengths of realist evaluation. This study enabled us to identify important contexts, understand how they shape medical students’ attitudes toward a career in psychiatry, and provide evidence-based recommendations that have strong potential for impact in real practice settings.

The final program theory reinforced elements of the existing published literature, as well as provided new insights. The seven most important contexts identified were a subset of those described previously: previous positive impact of mental health services on self, friends, or family [27]; exposure to multiple subspecialties [21]; previous positive experiences on placement in psychiatry [21, 23, 24, 27, 28]; a good work-life balance for psychiatry [29]; perceived danger from psychiatric patients [15]; perceived poor prognosis of patients [12–14]; and negative emotions evoked through work [16]. Interestingly, participants in our study did not view gender as an important context, in contrast to previous studies that highlighted women as more likely to form a positive attitude toward a career in psychiatry [25, 26]. This may relate to the UK context, where 59.6% of UK core psychiatry trainees were female from 2012 to 2017 [35], which also reflects the current gender split in the 2022 UK medical school intake demographics [34].

Previous studies have highlighted medical students’ negative perceptions toward psychiatry, including lack of an evidence base underpinning psychiatry [13, 14], perceived poor prognosis of patients [12–14], perceived danger from psychiatric patients [15], negative emotions evoked through work [16], and perceived low prestige [13]. These negative perceptions may develop from placement experiences, external work, negative media portrayal, or discussions with senior doctors. In this study, the negative perceptions deemed important by participants were perceived danger from psychiatric patients, perceived poor prognosis of patients, and negative emotions evoked through work. This could suggest that broader negative perceptions toward psychiatry as a specialty (e.g., lack of evidence base, low prestige) are changing, though we acknowledge that social desirability bias may have prevented students from divulging negatively viewed attitudes. Finally, previous international studies have identified that a lower income than other specialties forms negative attitudes toward a career in psychiatry [19]. Participants in this UK-based study did not recognize this context as important, although we recognized this might be an important context in other countries, particularly those that do not have a nationally funded health care system.

In line with previous findings, this study highlighted that previous positive experiences on placement, for example, encouragement from psychiatrists [27], good role models and teaching [22], well-organized placements [23], and students feeling involved [24], can shape positive attitudes toward a career in psychiatry. This study also highlighted the variety of what encompasses a positive experience, with different participants describing their positive experience differently. This highlights the challenge of creating placements that encourage career pursual for a variety of individuals. Despite the complexity of positive experience, this should be a target for change initiatives. This is emphasized in our study with aspects of positive experience spanning recommendations 1, 2, and 3. One example this study identified is that exposure to passionate and inspiring teachers in medical school could create more positive experiences on placement in psychiatry. Creating an environment that enables teachers to show passion is crucial to achieve this, such as a positive working environment, training in teaching, and dedicated time for teaching. Providing teaching fellows in psychiatry is one strategy that could facilitate this outcome.

There are few studies internationally that have explored the mechanisms that connect contexts to outcomes, such as inspiring students to make a difference [23], discomfort with psychiatry [14], a desire for flexibility and balance [29], fear of judgment [36], the familiarity of psychiatry [37], and tolerance of ambiguity [28]. This study showed that students could be inspired to make a difference and identified new, important mechanisms (e.g., feeling unable to make a difference; being concerned about emotional burden).

Significantly, by performing a realist evaluation, an assessment of the importance of contexts and mechanisms resulting in positive or negative attitudes toward a career in psychiatry was performed, enabling the production of a meaningful and trustworthy final program theory.

This study has multiple strengths. The research involved a purposefully sampled student group, ensuring a rich dataset that underpins the findings and recommendations, which enhances the study’s trustworthiness. In addition, by carefully implementing Manzano’s methodological guidance for realist interviews [33], this study provides novel insights that progress our understanding of this important problem and the possible solutions.

However, as with all studies, there are some limitations. The research involved one setting and one career stage, which provided a depth of understanding but means other important perspectives are less well understood. Given the voluntary nature of participation in this study, our study population had a higher percentage of females compared to the UK medical student population of that year, which may have influenced the findings. However, our interview participants did not feel that gender was an important context, and it is not part of the final program theory. Interestingly, more females volunteering to participate may indicate a higher percentage of females are considering a career in psychiatry, although this is out of the scope of this study.

Given the complexity of the interactions involved, certain mechanisms may have been inappropriately associated with contexts and outcomes. Although we anticipate that the CMOcs will be transferable to other similar contexts, further research will be needed to explore this, and we anticipate that tailoring of recommendations will be required for individual settings. Finally, the program theory considers attitudes rather than behaviors, but a positive attitude toward a career in psychiatry may not translate into increased psychiatry training program applications. However, we argue, as with the theory of planned behavior [10], that without a positive attitude a positive behavior is less likely to result.

Many of the contexts included in the initial program theory did not remain in the final program theory, although they may still be key for certain individuals, for example, having a family member who is a psychiatrist [27] and a background in the arts [28] were important for those who had that experience, but the experience was not widespread. Additionally, the two coauthors in psychiatry felt that a flattened medical hierarchy and the professional isolation of psychiatry were important contexts, but the interview participants did not view these as important, at least at their career stage. Participants did not need to have decided whether they would pursue a career in psychiatry, so there may be variance in the participants’ previous consideration toward this. We appreciate that those who have spent more time considering a career in psychiatry and those who have not may connect with differing parts of the final program theory, highlighting its adaptable and transferrable nature. These interesting perspectives could be followed up in future research, involving participants at a range of career stages (e.g., medical students, psychiatry trainees, psychiatry consultants). Future studies might also explore the strength intensity of attitudes toward a career in psychiatry. This was not possible in our study due to the challenge of quantifying qualitative data/responses.

We recommend that key stakeholders (e.g., policymakers and royal colleges, medical schools, passionate psychiatrists and educators) apply the recommendations of this study in their distinct contexts. Future studies could then examine the implementation of these recommendations and their translation to different countries and specialties, perhaps using realist methodologies. Additionally, a follow-up study might explore whether positive attitudes translated into changed behaviors through increased psychiatry training applications.

In conclusion, this realist evaluation advances understanding of how context shapes medical students’ attitudes toward a career in psychiatry and offers evidence-based recommendations that may have success in real-world settings. We conclude that stakeholders should provide dedicated mentors while on psychiatry placement, more psychiatry placements in the pre-clinical years of medical school, exposure to passionate and inspiring psychiatry teachers in medical school, and a focus on retaining interested students.

## Data Availability

Additional data that support the findings of this study are available from the corresponding author, LH, upon reasonable request.
